# Acute Jejunal Diverticulitis With Abscess Formation Complicated by Perforation: A Case Report

**DOI:** 10.7759/cureus.75129

**Published:** 2024-12-05

**Authors:** Meshael S Albahli, Shoag J Albugami, Nourah Alabdulaaly, Naif M Alshammari

**Affiliations:** 1 General Surgery, Prince Sultan Military Medical City, Riyadh, SAU; 2 Trauma and Acute Care Surgery, Prince Sultan Military Medical City, Riyadh, SAU

**Keywords:** abscess, acute diverticulitis, jejunal, laparotomy, perforation

## Abstract

Jejunal diverticulum perforation is a rare condition and presents diagnostic challenges. A 41-year-old male presented to the emergency room with a history of vague, generalized, and continuous abdominal pain for two days. He was vitally stable; however, the abdominal examination revealed a soft and distended abdomen with positive rebound tenderness. A computed tomography scan with contrast showed signs of a perforated proximal jejunal segment, along with adjacent pneumoperitoneum and a small fluid collection. The patient underwent diagnostic laparoscopy, which was converted to a midline laparotomy for further exploration of the small bowel. An 8-cm jejunal diverticulum with micro-perforation and abscess was identified during the procedure. The pathological diagnosis confirmed acute diverticulitis with abscess formation, and malignancy was ruled out. The patient remained stable throughout his postoperative recovery and was discharged on the sixth postoperative day.

## Introduction

Jejunal diverticulitis is a rare condition primarily affecting older adults, representing 0.3% to 1.3% of diverticulitis cases [1,2]. Its prevalence increases with age, particularly in males between their 60s and 80s [3]. While it can cause acute abdominal pain, clinical detection is challenging because patients often present with symptoms similar to other conditions, such as appendicitis, Crohn’s disease, or colonic diverticulitis [1]. Although jejunal diverticula are uncommon, they can lead to misleading clinical presentations, such as iron deficiency anemia due to diverticular bleeding. Furthermore, jejunal diverticula can present with severe complications such as perforation, hemorrhage, abscess, and obstruction [3,4].

Among the complications of jejunal diverticulosis, jejunal diverticulitis can often be treated conservatively with antibiotics rather than surgery [5]. In uncomplicated cases, conservative management may suffice [6]. However, most cases of complex jejunal diverticulosis require surgical intervention; without it, the mortality rate can be significant [7,8]. Early identification of the disease is crucial to prevent complications such as perforation. Because perforation and other consequences may arise if the disease is not identified early, it is imperative that practitioners be knowledgeable about this condition [8]. Surgery is usually performed as early as possible after diagnosis, as delays or missed diagnoses can lead to severe consequences [9]. Given that perforation carries an overall mortality rate of up to 40%, emergency laparoscopy is generally preferred over a conservative medical approach, with exploratory laparotomy, extensive lavage, segmental resection, and primary anastomosis being the standard treatment [2,10]. We presented a rare and challenging case of acute complicated jejunal diverticulitis with abscess formation and perforation.

## Case presentation

A 41-year-old male Saudi patient presented to the emergency department with a two-day history of abdominal pain. The pain was sudden, continuous, and diffuse, radiating to both flanks. It was accompanied by non-bilious, non-bloody nausea and vomiting, occurring multiple times a day, as well as one episode of melena three days before his presentation. The patient reported no fever, weight loss, night sweats, chest pain, cough, shortness of breath, or urinary issues.

**Figure 1:**
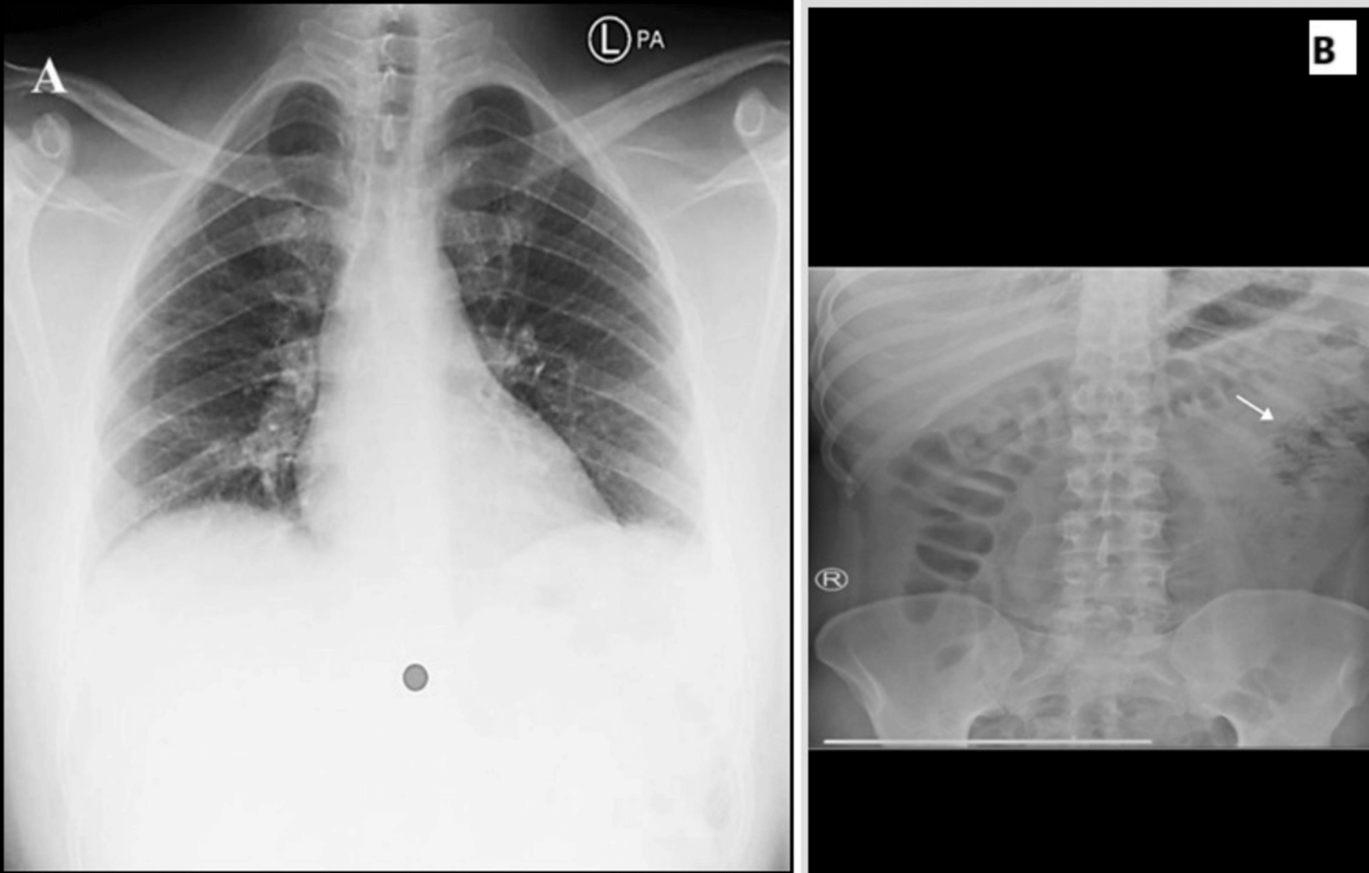
Chest postero-anterior (A) and abdominal (B) X-rays. An unremarkable chest and abdominal X-ray revealed a normal bowel loop diameter with Rigler’s sign.

**Figure 2:**
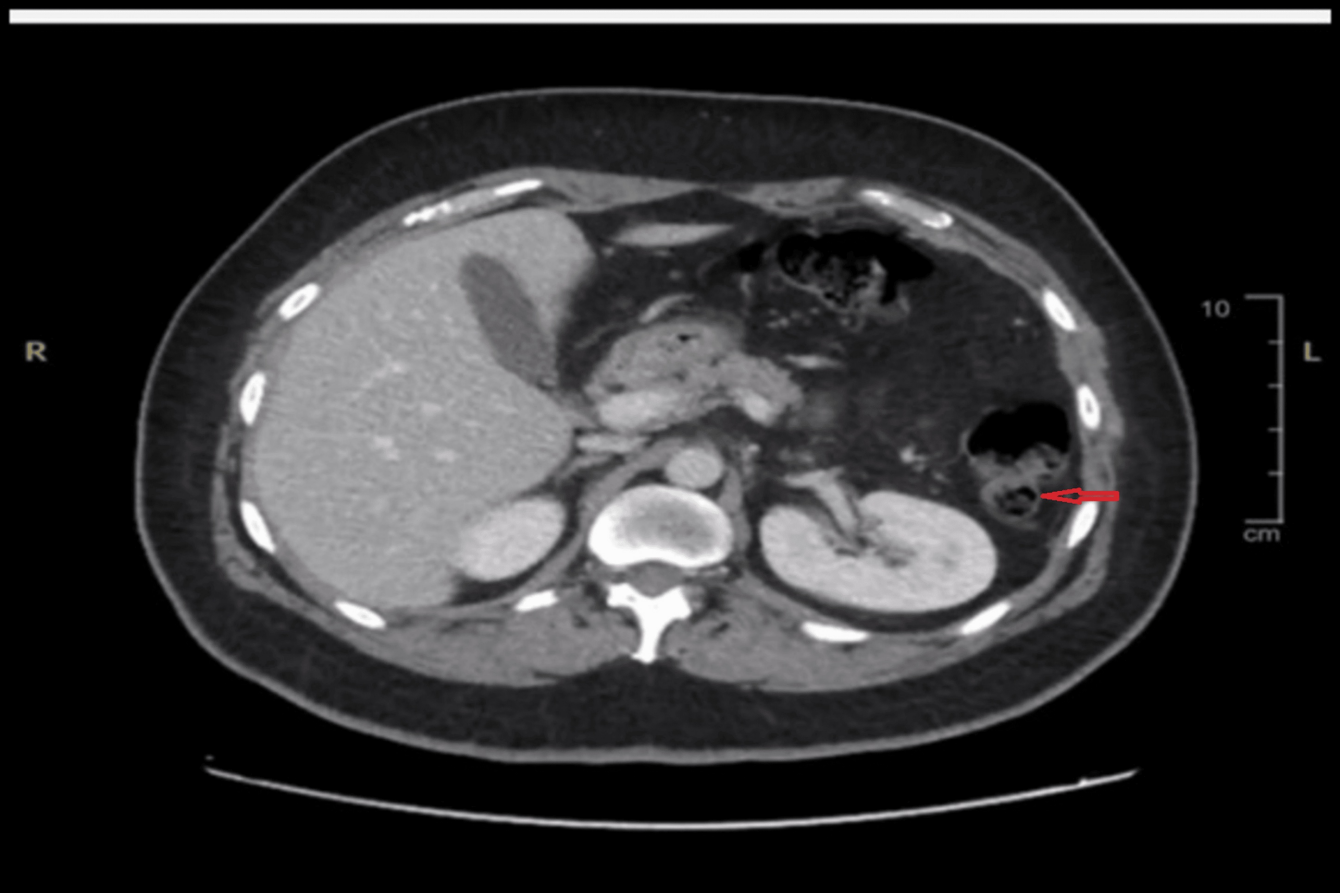
A computed tomography scan with contrast showing a focal diverticulum in the proximal/mid jejunal loop associated with diffuse wall thickening, significant surrounding fat stranding and adjacent pneumoperitoneum, and a small fluid collection.

Upon examination, his blood pressure was 125/69 mmHg, oral temperature was 37°C, heart rate was 74 bpm, respiratory rate was 18 breaths per minute, and oxygen saturation (SpO2) was 99%. He was conscious and alert. Abdominal examination revealed a soft but distended abdomen with positive rebound tenderness throughout his abdomen. He was admitted for further evaluation and observation. Blood tests showed normal results except for a slightly elevated white blood cell count (11.09 × 10^9^/L). Chest and abdominal radiographs (both supine and erect) are shown in Figure 1 and Figure 2. 

A computed tomography (CT) scan with contrast revealed a focal diverticulum in the proximal/mid jejunal loop associated with diffuse wall thickening and significant surrounding fat stranding. Adjacent pneumoperitoneum and a small fluid collection measuring approximately 2.3 × 1.5 × 2.3 cm were also present.

These findings suggested jejunal diverticulitis complicated by perforation. The remaining bowel loops appeared grossly unremarkable, with no signs of obstruction or ischemia.

**Figure 3:**
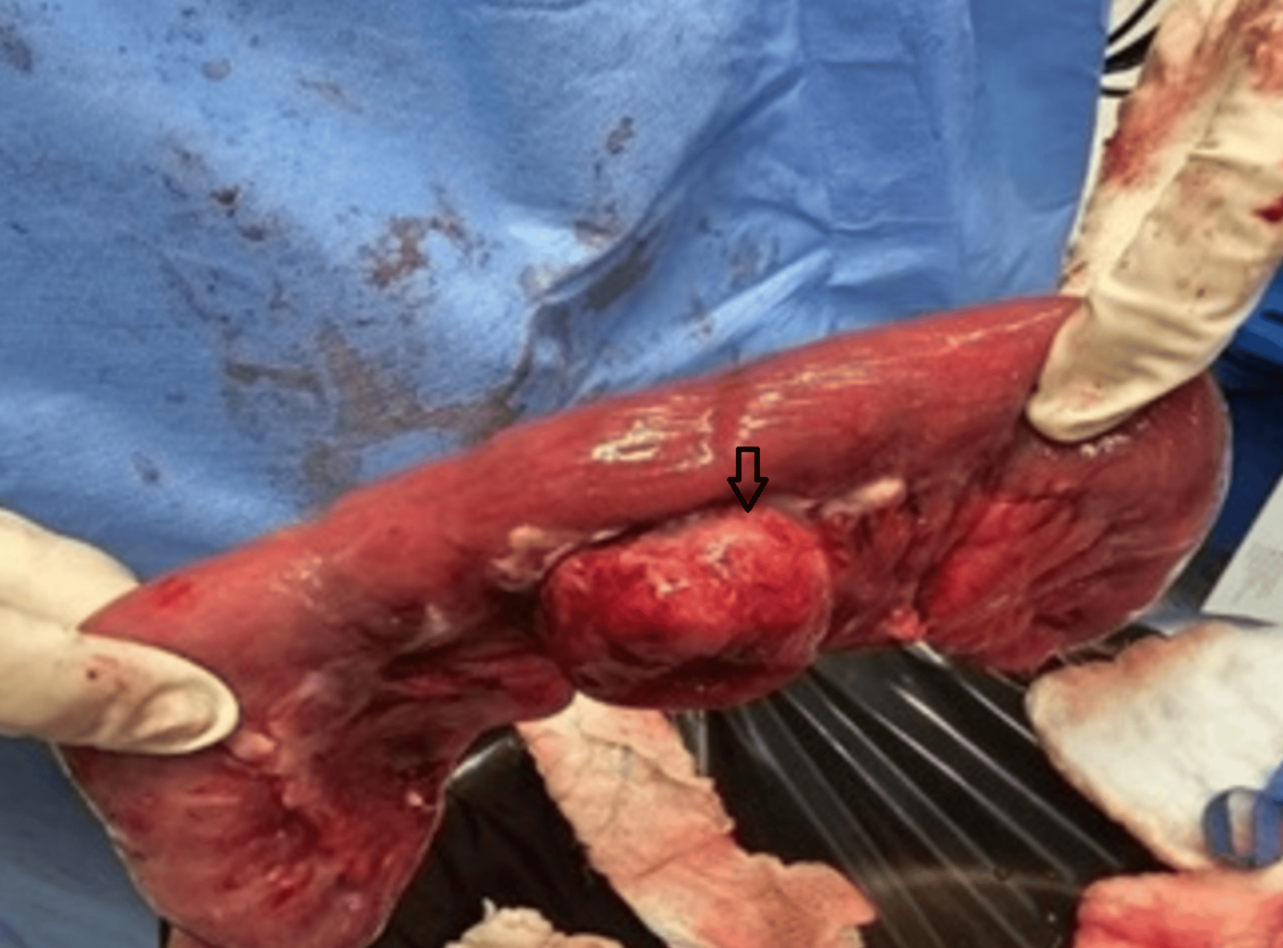
Surgical specimen of the diverticula approximately 50 cm from the duodenojejunal with micro-perforation and fibrin. It consists of a small bowel segment that is 19 cm long, 2 cm in open circumference, and 2 cm in fat extending from the wall. The serosa was gray-tan and covered by exudate, with remarkable tubal outpouching. The diverticulum was 8 cm from each margin (proximal and distal). There was a perforated area measuring 2 × 2 cm.

The surgical team decided on an emergency diagnostic laparoscopy. Under general anesthesia, a vertical umbilical incision was made using the open technique. The omentum showed an area of gas beneath two 5 mm ports inserted in the left lower quadrant, and midline exploration of the small bowel revealed an inflamed omentum and very large jejunal diverticula. The diagnostic laparoscopy was converted to a midline laparotomy to explore further the small bowel, which revealed a diverticulum approximately 50 cm from the duodenojejunal junction, showing micro-perforation and fibrin (Figure 3).

A small bowel resection of a 35 cm segment was performed between two staplers, along with wedge resection of the omentum and primary anastomosis. Two drains were placed: one at the anastomotic site and another in the pelvis. The surgical specimen consisted of a 19 cm segment of small bowel, 2 cm in circumference, with 2 cm of fat extending from the wall. The serosa was gray and covered with exudate, exhibiting prominent tubular outpouching. The diverticulum was located 8 cm from each margin (proximal and distal) and contained a perforated area measuring 2 × 2 cm.

Postoperatively, the patient was maintained on intravenous antibiotics (ceftriaxone and metronidazole), proton pump inhibitors, and anticoagulants (enoxaparin). He remained stable throughout his hospital stay, with serosanguinous output from both drains and normal laboratory results. He was discharged on the sixth postoperative day after drain removal, with instructions to follow up at the surgical outpatient department three weeks later. The pathological diagnosis indicated acute diverticulitis with abscess formation, and malignancy was ruled out. At his follow-up appointment, the patient reported being able to tolerate oral intake, passing bowel motions, and remaining hemodynamically stable. His abdomen was soft and lax, and the surgical clips were removed.

## Discussion

We presented a case of jejunal diverticulitis with abscess formation and significant tubular outpouching of unknown etiology. Similar to most cases of jejunal diverticulitis, our patient presented with abdominal pain [2-6]. While many cases are asymptomatic, jejunal diverticulitis can lead to severe and potentially fatal complications such as perforation, obstruction, and peritonitis. Symptoms may vary, including abdominal pain, gas distention, diarrhea, malabsorption, and steatorrhea [10]. Advances in surgical, pharmacological, and diagnostic techniques have reduced mortality rates, but preoperative detection of jejunal diverticulitis remains challenging [6]. If left untreated or improperly managed, bowel perforation can carry a mortality risk of up to 40% [10].

In elderly patients with small intestinal diverticulitis, the condition is often discovered incidentally, with many presenting with large, perforated, inflamed diverticula on the mesenteric side of the jejunum [11-13]. Furthermore, high-resolution imaging techniques frequently fail to confirm the diagnosis due to the location of the diverticula [2]. Abdominal CT is the preferred diagnostic method for identifying this condition, assessing its severity, and evaluating potential complications [1]. Upper gastrointestinal contrast studies may also be helpful for imaging this disease [3]. However, there is a lack of data regarding the presentation and treatment of small bowel diverticulitis, which raises concerns that patients with these symptoms may not receive appropriate diagnoses [3].

Therapeutic approaches vary based on disease severity and the patient's clinical condition [6]. Furthermore, treatment options depend on the symptoms and complications. Surgery is often necessary for perforations or when other medical interventions are ineffective [14]. Emergency surgery is recommended for perforation, cases with clinical or radiographic signs of peritonitis, failure of conservative treatment with worsening infection or sepsis, and persistent diverticular bleeding that cannot be controlled by radiological or endoscopic methods [15]. The standard of care typically includes exploratory laparotomy, extensive lavage, segmental resection, and primary anastomosis [10]. In cases of complicated jejunal diverticulitis, emergency laparoscopy is preferred over a conservative treatment approach [2].

## Conclusions

Jejunal diverticulitis should be included in the differential diagnosis for patients presenting with acute abdomen. While jejunal diverticula are relatively uncommon, this condition can lead to significant complications, including perforation. Perforation, although rare, presents considerable diagnostic challenges because the symptoms can overlap with those of other acute abdominal conditions.

Failure to accurately diagnose jejunal diverticulitis can have serious consequences, including a high mortality rate. This underscores the importance of thorough clinical evaluation and consideration of jejunal diverticulitis in patients presenting with acute abdominal pain. Timely intervention is crucial; a high index of suspicion can facilitate prompt imaging and treatment, potentially reducing morbidity and mortality associated with complications such as perforation or abscess formation. Ultimately, increased awareness among surgeons and emergency room physicians about the existence and implications of jejunal diverticulitis is essential for improving patient outcomes in acute abdominal scenarios.
